# Successful Treatment of Postmenopausal Exacerbation of Abdominal Lymphangioleiomyomatosis With Sirolimus: A Report of a Rare Case

**DOI:** 10.7759/cureus.69549

**Published:** 2024-09-16

**Authors:** Kazuki Uchida, Kazunori Tobino

**Affiliations:** 1 Respiratory Medicine, Iizuka Hospital, Iizuka, JPN

**Keywords:** abdominal lymphangioleiomyomas, lymphangioleiomyomatosis, mtor, postmenopausal progression, sirolimus

## Abstract

Lymphangioleiomyomatosis (LAM) is a rare disease that primarily affects women of childbearing age and often stabilizes after menopause. We report an unusual case of LAM progression in a 70-year-old postmenopausal woman. Diagnosed with LAM at age 53, the patient experienced progression of abdominal lesions 17 years post-diagnosis, despite stable pulmonary function. Notably, she was receiving aromatase inhibitors for breast cancer. Abdominal CT and MRI revealed enlarging nodules, while serum vascular endothelial growth factor-D (VEGF-D) levels were elevated. Treatment with sirolimus (2 mg/day, later adjusted) led to significant improvement in symptoms and a reduction in abdominal nodules. This case highlights the potential for LAM progression in postmenopausal women, primarily manifesting as abdominal lesions. It underscores the importance of long-term follow-up in LAM patients, regardless of menopausal status, and demonstrates the efficacy of sirolimus in managing postmenopausal LAM progression. Further research is needed to understand the mechanisms driving LAM activity in the absence of high estrogen levels and to optimize treatment strategies for this patient population.

## Introduction

Lymphangioleiomyomatosis (LAM) is a slowly progressive, systemic, intractable disease characterized by the proliferation of smooth muscle-like LAM cells in the lungs and axial lymph nodes, forming multiple cysts in the lungs [1]. It is classified into tuberous sclerosis complex (TSC)-LAM, associated with TSC, and sporadic LAM, which occurs alone [2]. The prevalence of LAM is three to eight per million persons in seven countries, including the U.S. and Japan [3]. The most common symptoms of LAM are progressive dyspnea on exertion and spontaneous pneumothorax [4]. The average age at diagnosis of LAM is around 35 years, and the disease was traditionally thought to affect young women of childbearing age [5]. Estrogen has a strong influence on the development of LAM, and the disease often stabilizes after menopause [6]. However, rare cases of LAM developing or worsening after menopause have been reported, the mechanism of which is unclear. We herein report a case of LAM with postmenopausal exacerbation of abdominal lesions.

## Case presentation

A 70-year-old Japanese woman was referred to our department for a detailed examination and treatment of her abdominal nodules. She underwent a partial left mastectomy and sentinel node biopsy for left breast cancer at age 55 years. Postoperatively, the patient was started on radiation therapy and aromatase inhibitors for five years. At age 64, she underwent preoperative chemotherapy for right human epidermal growth factor receptor 2 (HER2)-positive breast cancer with fluorouracil, epirubicin hydrochloride, and cyclophosphamide hydrochloride for six cycles. In addition, four cycles of paclitaxel and trastuzumab were administered. At age 65, she underwent a partial right mastectomy and sentinel node biopsy. After the surgery, the patient received radiation therapy to the preserved breast and was administered trastuzumab. There were no oral medications or supplements that elevated estrogen.

**Figure 1:**
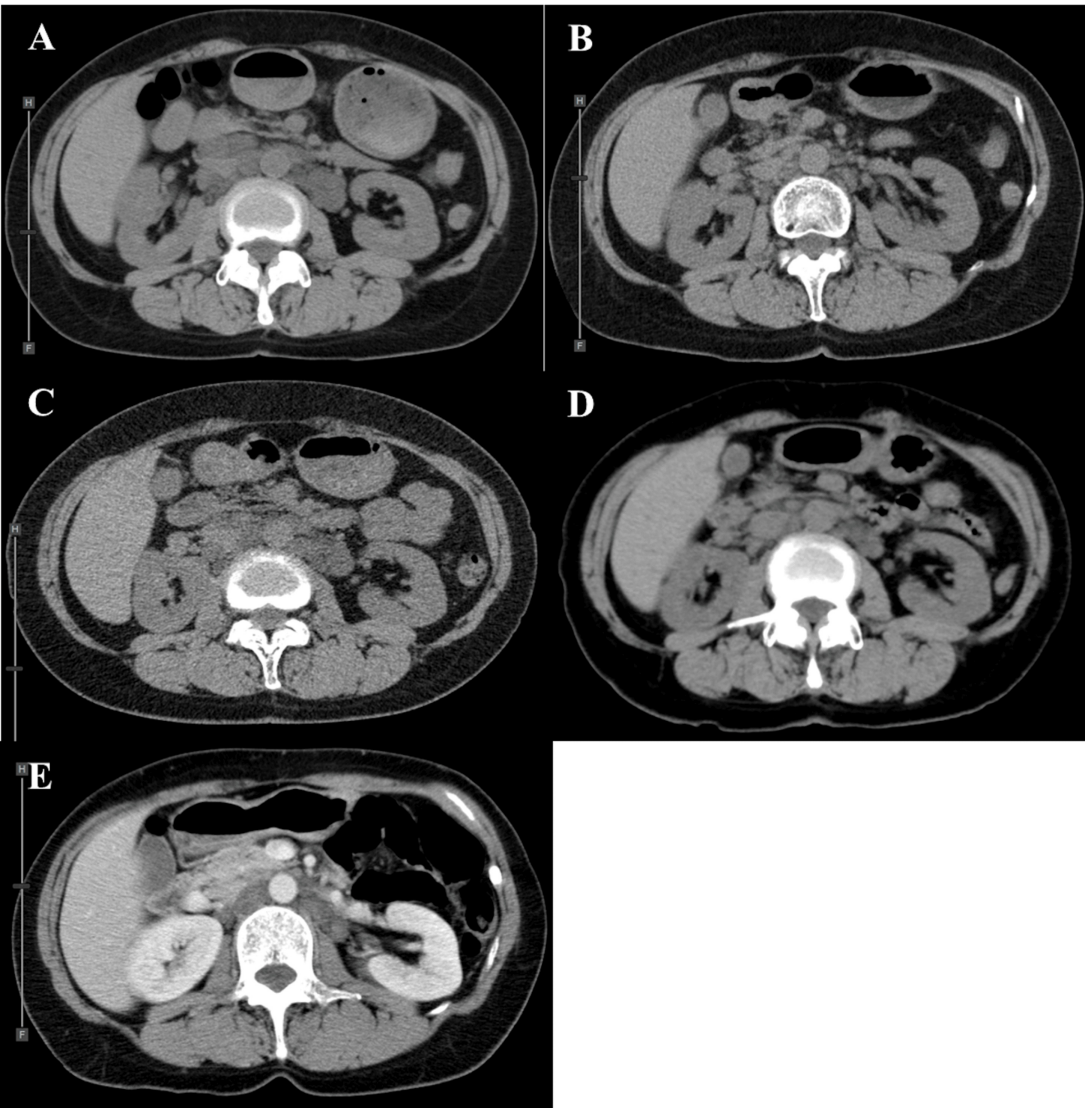
Changes in CT findings of the abdominal lesion over time prior to presentation at our hospital. (A) Initial findings when the abdominal lesion was first identified at another hospital (seven years before presentation); (B) Findings six years before presentation at our hospital: the abdominal lesion had decreased in size; (C) Findings five years before presentation at our hospital: the abdominal lesion had slightly increased in size; (D) Findings four years before presentation at our hospital: the abdominal lesion had decreased in size again; (E) Findings three years before presentation at our hospital: the abdominal lesion had increased in size again.

At the age of 53, she was diagnosed with LAM via a surgical lung biopsy, after a chest computed tomography (CT) performed for a detailed examination of her breast cancer incidentally revealed multiple lung cysts. Pulmonary function tests at the time of diagnosis were normal, with a forced expiratory volume in one second (FEV1) of 2.46 liters, which was 119.4% of the predicted value, and since there were no subjective symptoms and the abdominal lesions were mild, the patient was followed up without treatment. A body CT taken at the previous hospital 10 years after diagnosis for follow-up showed no changes in multiple pulmonary cysts, but new multiple nodules appeared around the aorta and bilateral common iliac arteries. However, she had no subjective symptoms and was placed under observation. Thereafter, the enlarged lymph nodes showed spontaneous shrinkage and relapse (Figures 1A-1D).

Three years later, she presented to her previous doctor with the onset of right upper quadrant pain. Upper and lower gastrointestinal endoscopy did not reveal the cause of her pain, and abdominal CT showed an increase in the number and size of multiple small nodules around the abdominal aorta and inferior vena cava, rather than a single large mass (Figure 1E). Differential diagnoses for the patient's right upper abdominal pain included cholelithiasis, cholecystitis, peptic ulcer disease, gastroesophageal reflux disease (GERD), and hepatitis. These conditions were ruled out through upper and lower gastrointestinal endoscopy and abdominal imaging. Given the patient's history of LAM and the concurrent enlargement of abdominal nodules, the pain was attributed to the progression of LAM-associated lymphangioleiomyomas. Abdominal MRI showed multifocal nodules contiguous with high signal intensity on T2-weighted images and low signal intensity on T1-weighted images. The abdominal mass consisted of multiple fused nodules, each measuring 1-3 cm in length. These nodules extended in the axial direction of the body from the upper to lower abdomen, forming a confluent mass along the retroperitoneal space. The patient had been prescribed analgesic medication by her previous physician when she complained of right upper abdominal pain three years before coming to our hospital. This medication provided temporary relief and the pain subsided for several months. However, over the next eight months, the symptoms recurred and gradually worsened, so the previous doctor increased the dose of analgesics and added an antispasmodic. Despite these adjustments, her abdominal pain continued to worsen and she was referred to our hospital for further assessment and management.

**Figure 2:**
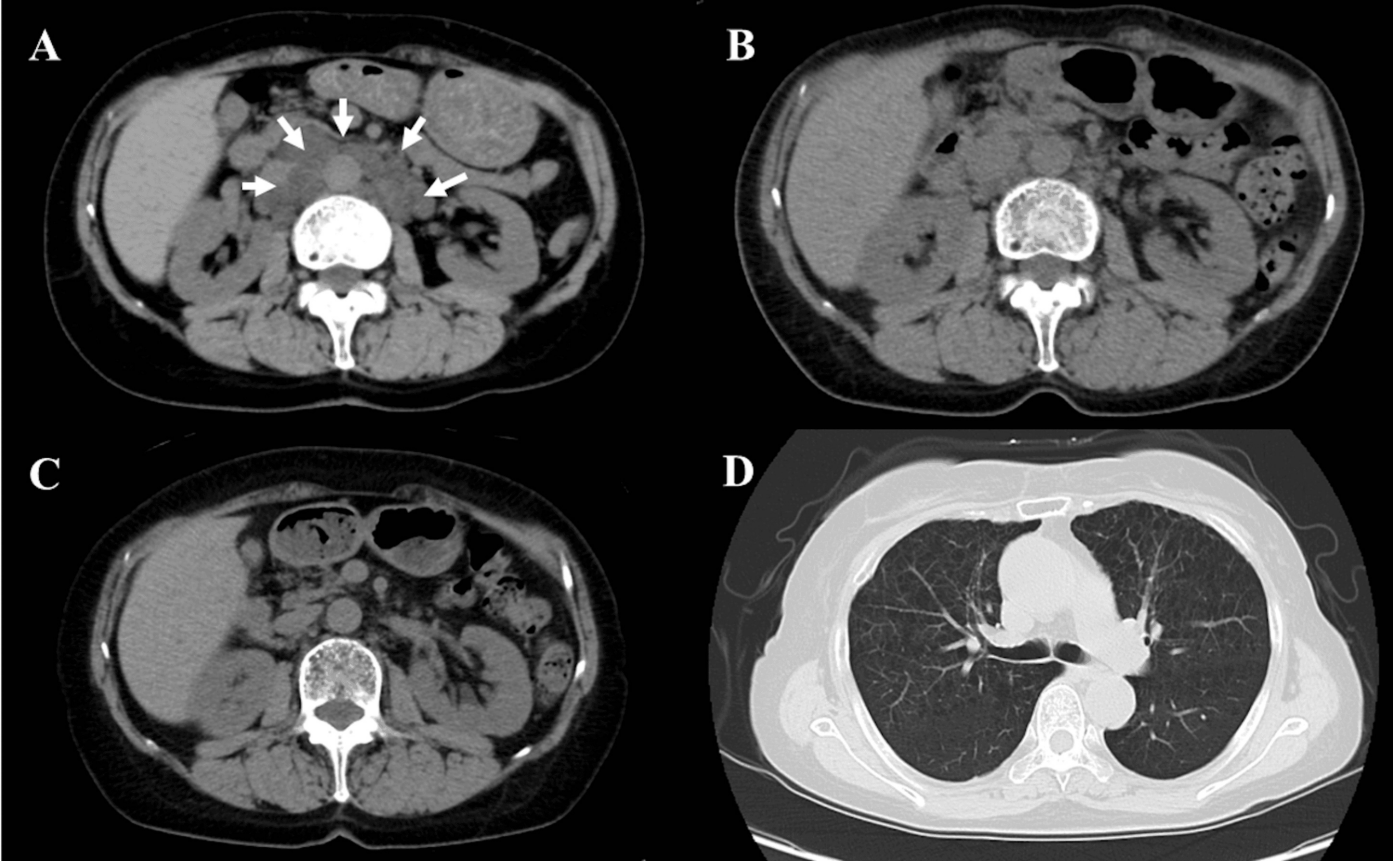
Abdominal and pulmonary CT images over time after presentation to our hospital. CT images at the initial visit (A) showed an increase and enlargement of multiple abdominal nodules (arrows). A CT image taken two months after starting medication (B) revealed a reduction in the size of the abdominal nodules. After nine months of medication (C), the abdominal nodules had nearly disappeared. CT imaging of the pulmonary lesions (D) showed no significant changes throughout the disease course.

On examination, her level of consciousness was clear, blood pressure was 162/58 mmHg, pulse rate was 58 beats per minute, body temperature was 36.1°C, respiratory rate was 16 breaths per minute, and oxygen saturation (SpO2) measured by pulse oximetry was 98% while the patient was breathing ambient air. The only subjective symptom was lower abdominal pain. Physical examination revealed the abdomen was flat and soft, with no tenderness. Blood tests showed no significant findings except a mildly elevated C-reactive protein (CRP) of 1.90 mg/dL. Prolactin was low at 6.20 ng/mL, and estradiol and progesterone were below the measurement sensitivity. Serum vascular endothelial growth factor-D (VEGF-D) was elevated at 1382.9 pg/mL. Laboratory tests revealed that the patient's estradiol levels were below the detection limit (<10 pg/mL), confirming her postmenopausal status and ruling out potential exogenous estrogen sources. A detailed dietary history was obtained, revealing that the patient maintained a balanced diet without excessive consumption of high-fat foods. She denied taking any supplements, including those with potential estrogenic effects such as soy isoflavones or black cohosh. The patient's dietary habits and supplement intake were deemed unlikely to contribute to her condition. Abdominal CT and ultrasonography confirmed the enlargement of the intra-abdominal nodules previously noted by her former doctor (Figure 2A).

Chest CT showed multiple pulmonary cysts (Figure 2D). There were no respiratory symptoms during treatment at our department, and pulmonary function tests showed a forced vital capacity (FVC) of 3.43 liters (148.5% of the predicted value), an FEV1 of 2.08 liters (113.7% of the predicted value), and a diffusing capacity of the lung for carbon monoxide (DLCO) of 9.81 mL/min/mmHg (65.8% of the predicted value). The pulmonary function test performed three years before the patient's visit at the previous hospital showed an FVC of 3.24 liters (140.9% of the predicted value), an FEV1 of 2.03 liters (116.7% of the predicted value), which was similar to the result obtained at our department. DLCO was not measured and could not be compared.

Based on the above results, especially the imaging findings and the serum VEGF-D results, a diagnosis of exacerbation of abdominal lymphangioleiomyoma was made. Given the patient's history of breast cancer, the possibility of metastases or recurrence was carefully considered. However, the abdominal lesions observed on CT scans from the previous hospital showed near-water density low attenuation, lower than that of typical lymph nodes. Furthermore, the location of these lesions was atypical for breast cancer recurrence or metastasis. These imaging characteristics strongly suggested LAM-related abdominal involvement rather than malignancy.

Based on these findings, along with the elevated serum VEGF-D levels which are highly specific for LAM, we decided not to perform additional tests such as tumor marker analysis or positron emission tomography (PET)-CT scans. Instead, we opted to confirm the diagnosis of LAM based on the serum VEGF-D levels and the response to sirolimus treatment. This approach is consistent with current diagnostic guidelines for LAM, which recognize serum VEGF-D as a diagnostic biomarker and acknowledge that biopsy may not always be necessary when clinical and radiological findings are characteristic. Sirolimus (2 mg/day) treatment was started, and the abdominal pain disappeared after one month of treatment. There was a mild decrease in white blood cells of 2,820/µL, and the blood level of sirolimus was as high as 11.0 ng/mL. Therefore, the dose of sirolimus was reduced to 1 mg as it was judged to be an adverse reaction. Two months after the start of treatment, abdominal CT showed nodule reduction. Although the sirolimus blood level was low (3.6 ng/mL), blood tests showed mild neutropenia (1,240/µL), so the sirolimus dose was changed to 1 mg every other day. Six months after the start of treatment, the patient's pain flared up, so the dose of sirolimus was increased again to 1 mg/day. Her symptoms did not recur thereafter, and a CT scan nine months later showed no re-enlargement of the abdominal nodules (Figures 2B, 2C).

## Discussion

This case report highlights a unique instance of LAM exacerbation in a postmenopausal woman, characterized by the development and progression of abdominal nodules. Our patient, diagnosed with LAM at the age of 53, exhibited stable disease for a decade before experiencing significant abdominal mass growth and pain postmenopause. Treatment with sirolimus resulted in substantial symptomatic relief and mass reduction, underscoring the potential of sirolimus as an effective therapeutic agent for abdominal LAM lesions.

LAM typically affects women of childbearing age, with an average diagnosis age of 35 years [5]. The disease is strongly influenced by estrogen and often stabilizes after menopause [6]. However, there are rare reports of postmenopausal LAM progression involving abdominal nodules. For example, Bhattacharyya and Balogh described an 89-year-old woman with a mass extending from the pelvic cavity to the retroperitoneum [7]. In another case of a premenopausal woman, Wong et al. reported a 32-year-old woman with abdominal pain associated with an abdominal LAMT lesion [8]. These cases and ours demonstrate that abdominal lesions in postmenopausal LAM patients are rare but present and that abdominal lesions can be symptomatic. Our case is particularly notable for the postmenopausal exacerbation of abdominal lesions without concurrent pulmonary function deterioration.

The pathophysiological mechanisms behind LAM exacerbation postmenopause are not fully understood. Estrogen is known to promote LAM cell proliferation, and its decline postmenopause typically stabilizes the disease [6]. However, our patient's condition worsened postmenopause despite low estrogen levels, suggesting other factors at play. The patient was on aromatase inhibitors for breast cancer, which further reduced estrogen levels. The observed LAM progression despite suppressed estrogen levels points towards potential genetic or molecular alterations within LAM cells or the involvement of other hormonal or growth factor pathways, such as VEGF-D, which was elevated in this patient.

The efficacy of sirolimus for LAM has been demonstrated in the Multicenter International Lymphangioleiomyomatosis Efficacy and Safety (MILES) study, showing beneficial effects including stabilization of FEV1, improvement of FVC, and quality of life [9]. An intriguing aspect of this case is the presence of abdominal pain associated with retroperitoneal lymphangioleiomyomas. Typically, LAM patients with retroperitoneal involvement, even those with larger lesions, often remain asymptomatic. The mechanism behind why these lesions caused significant pain in our postmenopausal patient remains unclear. Several hypotheses can be considered: (1) Hormonal changes: The postmenopausal state might alter the microenvironment of LAM cells, potentially increasing their inflammatory properties or sensitivity of surrounding tissues; (2) Nerve compression: Even small nodules, if strategically located, might compress or irritate nearby nerves, causing disproportionate pain; (3) Lymphatic obstruction: Progressive growth of lymphangioleiomyomas, even if small, might cause localized lymphatic obstruction, leading to tissue edema and pain; (4) Altered pain perception: Age-related changes in pain perception or concurrent conditions might lower the pain threshold. Further research is needed to elucidate the precise mechanisms of pain generation in postmenopausal LAM patients with retroperitoneal lesions. This case underscores the importance of considering LAM in the differential diagnosis of abdominal pain in postmenopausal women, even when imaging shows only small nodules. The retrospective analysis and lack of longitudinal data on the patient's hormone levels and molecular markers limit the ability to draw definitive conclusions about the underlying mechanisms. The mechanism of abdominal lesions associated with LAM is not precise. Physical defects and compression of abdominal organs may also be a member. There are also reports that biliary ascites related to obstruction of lymphatic vessels may cause abdominal pain. Further investigation is needed [10]. Additionally, while sirolimus showed effectiveness in our patient, the optimal dosing regimen and long-term outcomes require further validation through larger clinical studies. The MILES trial indicated that discontinuation of sirolimus led to a decline in FEV1, suggesting the need for ongoing treatment to prevent tumor re-growth [11].

## Conclusions

In conclusion, this case highlights the rare but significant possibility of LAM exacerbation in postmenopausal women, manifesting primarily through abdominal nodule progression. The effective use of sirolimus in this context provides valuable insight into potential management strategies for similar cases. Continued research is crucial to understand the factors driving LAM activity in postmenopausal patients and to develop targeted therapies that can address these unique clinical scenarios. This case emphasizes the need for vigilance in monitoring postmenopausal LAM patients for potential disease progression, even in the absence of estrogen. Additionally, while aromatase inhibitors are potential therapeutic agents for LAM, their role and effectiveness in postmenopausal patients warrant further investigation.
